# The nutrient distribution in the continuum of the pericarp, seed coat, and kernel during *Styrax tonkinensis* fruit development

**DOI:** 10.7717/peerj.7996

**Published:** 2019-10-31

**Authors:** Qikui Wu, Zihan Zhang, Huan Peng, Yali Wu, Fangyuan Yu

**Affiliations:** 1 Nanjing Forestry University, Collaborative Innovation Center of Sustainable Forestry in Southern China, College of Forest Science, Nanjing, Jiangsu, China; 2 Chinese Academy of Forestry, State Key Laboratory of Tree Genetics and Breeding & Key Laboratory of Tree Breeding and Cultivation, State Forestry Administration, Research Institute of Forestry, Beijing, China; 3 Gaochun District Agricultural and Rural Bureau, Nanjing, Jiangsu, China

**Keywords:** *Styrax tonkinensis*, Nutrient distribution, Enzyme activity, Continuum, Fruit development

## Abstract

**Background:**

*Styrax tonkinensis* is a great potential biofuel as the species contains seeds with a particularly high oil content. Understanding the nutrient distribution in different parts of the fruit is imperative for the development and enhancement of *S. tonkinensis* as a biodiesel feedstock.

**Methods:**

From 30 to 140 days after flowering (DAF), the development of *S. tonkinensis* fruit was tracked. The morphology change, nutrient content, and activity of associated enzymes in the continuum of the pericarp, seed coat, and kernel were analyzed.

**Results:**

Between 30 and 70 DAF, the main locus of dry matter deposition shifted from the seed coat to the kernel. The water content within the pericarp remained high throughout development, but at the end (130 DAF later) decreased rapidly. The water content within both the seed coat and the kernel consistently declined over the course of the fruit development (30–110 DAF). Between 70 and 80 DAF, the deposition centers for sugar, starch, protein, potassium, and magnesium was transferred to the kernel from either the pericarp or the seed coat. The calcium deposition center was transferred first from pericarp to the seed coat and then to the kernel before it was returned to the pericarp. The sucrose to hexose ratio in the seed coat increased between 30 and 80 DAF, correlating with the accumulation of total soluble sugar, starch, and protein. In the pericarp, the sucrose to hexose ratio peaked at 40 and 100 DAF, correlating with the reserve deposition in the following 20–30 days. After 30 DAF, the chlorophyll concentration of both the pericarp and the seed coat dropped. The maternal unit (the pericarp and the seed coat) in fruit showed a significant positive linear relationship between chlorophyll b/a and the concentration of total soluble sugar. The potassium content had significant positive correlation with starch (ρ = 0.673, *p* = 0.0164), oil (ρ = 0.915, *p* = 0.000203), and protein content (ρ = 0.814, *p* = 0.00128), respectively. The concentration of magnesium had significant positive correlation with starch (ρ = 0.705, *p* = 0.0104), oil (ρ = 0.913, *p* = 0.000228), and protein content (ρ = 0.896, *p* = 0.0000786), respectively. Calcium content had a significant correlation with soluble sugar content (ρ = 0.585, *p* = 0.0457).

**Conclusions:**

During the fruit development of *S. tonkinensis*, the maternal unit, that is, the pericarp and seed coat, may act a nutrient buffer storage area between the mother tree and the kernel. The stage of 70–80 DAF is an important time in the nutrient distribution in the continuum of the pericarp, seed coat, and kernel. Our results described the metabolic dynamics of the continuum of the pericarp, seed coat, and kernel and the contribution that a seed with high oil content offers to biofuel.

## Introduction

Fruit, as the reproductive organ of higher plants, has high sink strength. During fruit development, large amounts of nutrients are transferred from the mother tree into the continuum of the pericarp, seed coat, and kernel by vascular tissue (Ruan et al., 2012; Zhang et al., 2005). Fruit can be divided into two broad categories based on the shape (water content) of the pericarp. The first category, fleshy fruit, is characterized by the development of the pericarp and accessory parts into succulent tissues, as in benzoin (Zhang et al., 2018a). The second category is dry fruit, in which the entire pericarp becomes dry at maturity (Pabon-Mora & Litt, 2011). Most research about fruit development has focused on fleshy fruit, specifically that of horticultural plants such as olive, citrus, and Siberian apricot. These plants store carbon and nitrogen in their swollen pericarp (Conde, Delrot & Gerós, 2008; Li et al., 2009; Wang et al., 2019). Fruit with an inedible pericarp houses the main storage of photosynthetic products in its seed. There is very little research on these seeds.

The pericarp and the seed coat are the maternal layers surrounding the kernel that produce and accumulate desired molecules and regulate nutrient transport to the kernel during seed development on the mother tree (Sano et al., 2016; Li et al., 2018a). They also have other functions such as providing chemical and mechanical protection as well as defining final seed size and shape (Francoz, Lepiniec & North, 2018; Li et al., 2018b). *Styrax tonkinensis* (Pierre) Craib ex Hartwich, widely distributed in the south provinces in China, has great potential as a biodiesel species as it has a seed kernel with high oil content (nearly 60%), excellent fatty acid (FA) composition (high oleic and linoleic acid contents), and good fuel properties which satisfies the biodiesel standards of China (GB/T 20828), the European Union (EN 14214), Germany (DIN V51606), and the USA (ASTM D6751) (Zhang et al., 2018a; Wu et al., 2019). Its pericarp and seed coat stay green for up to 5 months during reproductive development, while the kernel turns milky-white and enlarges considerably and the kernel oil content showed an up-down-up trend with high contents (Zhang et al., 2017). Therefore, the continuum of the pericarp, seed coat, and kernel may keep metabolic processes and substance transport active during *S. tonkinensis* fruit development. For oil seeds, the studies of nutrients were mostly focused on the oil accumulation in the pericarp or kernels. There is little information on the nutrient distribution and nutritional management of the continuum of the pericarp, seed coat, and kernel.

In this study, reserve accumulation and the activity of the main associated metabolic enzymes of the continuum of the pericarp, seed coat, and kernel were measured during fruit development in *S. tonkinensis*. We wanted to answer the following questions: (1) How does the continuum, especially the pericarp and the seed coat, change during *S. tonkinensis* fruit development? (2) As the connecting carrier of the mother tree and the kernel, what roles do the pericarp and the seed coat play in nutrient accumulation of the kernel? In addition, the pericarp and the seed coat stay green for 5 months during fruit development, so (3) what effect does the change in chlorophyll content have on carbon competition in the kernel? Answers to these questions may help us to further understand the metabolic dynamics during seed development in *S. tonkinensis* and may provide reference to control the oil content of biofuel seeds.

## Materials and Methods

### Plant materials

Five-year-old *S. tonkinensis* trees (of Jishui, Jiangxi provenance) were planted in the *Styracaceae* Germplasm Repository, Luhe District, Nanjing, China (32°54′N, 118°50′E) where they grew without the use of fertilizer. In late May, 15 trees were tagged for sampling. Since June 23rd, 30 days after flowering (DAF), fresh fruits were randomly harvested from branches pointing in different directions from each selected plant. A 9-day interval elapsed between each harvest. Drupes were sealed in plastic bags immediately and then embedded in an insulation ice box (−10 °C). The pericarp, seed coat, and kernel from 30 fresh fruits, in four replicates each, were weighed just after they had been carefully separated using a clean dissecting needle and blades (fresh matter (FM)) and then were weighed again after drying at 65 °C for 72 h (dry matter (DM)). The remaining fruits were immediately frozen in liquid nitrogen and stored at −80 °C for other analyses.

### Fruit morphology

For each treatment, 50 fresh fruits were randomly selected from each developmental stage. The fruit lengths (*L*_f_) and widths (*W*_f_) and seed lengths (*L*_s_) and widths (*W*_s_) were measured with a digital auto caliper. The fruit (*V*_f_) and seed (*V*_s_) volumes and the surface area of the fruit (*S*_f_) and seed (*S*_s_) were determined according to the following equations:

(1) $${V_{\rm{f}}}({\rm{m}}{{\rm{m}}^3}) = {{4\pi } \over 3} \times ({{{L_{\rm{f}}}} \over 2} \times {{{W_{\rm{f}}}} \over 2} \times {{{W_{\rm{f}}}} \over 2}) = {\pi  \over 6}{L_{\rm{f}}}{W_{\rm{f}}}^2$$

(2) $${V_{\rm{s}}}({\rm{m}}{{\rm{m}}^3}) = {{4\pi } \over 3} \times ({{{L_{\rm{s}}}} \over 2} \times {{{W_{\rm{s}}}} \over 2} \times {{{W_{\rm{s}}}} \over 2}) = {\pi  \over 6}{L_{\rm{s}}}{W_{\rm{s}}}^2$$

(3) $${S_{\rm{f}}}({\rm{m}}{{\rm{m}}^2}) = {{4\pi } \over 3}({{{L_{\rm{f}}}} \over 2} \times {{{W_{\rm{f}}}} \over 2} + {{{L_{\rm{f}}}} \over 2} \times {{{W_{\rm{f}}}} \over 2} + {{{W_{\rm{f}}}} \over 2} \times {{{W_{\rm{f}}}} \over 2}) = {\pi  \over 3}(2{L_{\rm{f}}}{W_{\rm{f}}} + {W_{\rm{f}}}^2)$$

(4) $${S_{\rm{s}}}({\rm{m}}{{\rm{m}}^2}) = {{4\pi } \over 3}({{{L_{\rm{s}}}} \over 2} \times {{{W_{\rm{s}}}} \over 2} + {{{L_{\rm{s}}}} \over 2} \times {{{W_{\rm{s}}}} \over 2} + {{{W_{\rm{s}}}} \over 2} \times {{{W_{\rm{s}}}} \over 2}) = {\pi  \over 3}(2{L_{\rm{s}}}{W_{\rm{s}}} + {W_{\rm{s}}}^2)$$

### Carbohydrate content

To measure soluble sugar and starch content of different fruit parts (i.e., the pericarp, seed coat, and kernel) by UV/VIS spectrophotometer (DU-800; Beckman Coulter Inc., Brea, CA, USA), 500 mg fresh samples (at 30 and 40 DAF, 50 kernels were subsampled and weighed; this same procedure was followed for all analyses of the samples) were ground and heated on the boiling water bath for 20 min. After centrifuging at 5,000×*g* rpm for 10 min at 15 °C twice, the supernatant was used for sugar analysis and the sediment for starch content measurement. Total soluble sugar content in each sample was measured using an anthrone colorimetric method with glucose as a standard at 630 nm (Li, 2006). The fructose, glucose, and sucrose content of the pericarp and the seed coat were quantified according to Zhang et al. (2017). The starch content was calculated through a standard curve prepared using a starch solution of various concentrations at 630 nm (Li, 2006).

### Total free amino acid and total protein content

A total of 500 mg fresh samples of the pericarp, seed coat, and kernel were ground in an ice bath and homogenized in five mL 10% (v/v) acetic acid and then centrifuged at 5,000×*g* rpm for 10 min at 15 °C. The supernatant was diluted with deionized water to 100 mL and used for the quantification of total free amino acid content at 570 nm via UV/VIS spectrophotometer according to Rosen (1957) and following the procedure of Li (2006), with leucine as the standard.

Total soluble protein content was quantified according to Bradford (1976). A total of 500 mg fresh samples were homogenized in a five mL extraction buffer containing a 0.02 mol/L phosphate buffer (PBS, pH 7.5). After centrifuging at 6,000×*g* rpm for 20 min at 15 °C, the supernatant was diluted properly before being measured against bovine serum albumin as the standard at 595 nm via UV/VIS spectrophotometer.

### Chlorophyll content

Chlorophyll content of the pericarp and seed coat was determined upon extraction in buffered aqueous 80% acetone via UV/VIS spectrophotometer (Li, 2006; Porra, 2002). A total of 500 mg fresh samples were ground with some quartz sand and calcium carbonate powder and homogenized in 80% acetone. The samples were transferred to a 25 mL volumetric flask with 80% acetone. Absorbance at 646 and 663 nm of the supernatant was measured with a spectrophotometer to conduct the chlorophyll a (Chl a) and chlorophyll b (Chl b) calculation.

### Potassium, calcium, and magnesium content

The potassium, calcium, and magnesium content of the pericarp, seed coat, and kernel were measured by flame atomic absorption spectrophotometry (model AA200; PerkinElmer, Waltham, MA, USA) (Garcia-Alegria et al., 2018). A total of 500 mg fresh samples were placed in a kay type bottle with nine mL concentrated sulfuric acid and one mL perchlorate. The samples were left overnight before the heating digestion of 30 min, then filtered and then moved into a 100 mL volumetric flask and brought to volume with deionized water. The standard curves and fire effect parameters (potassium: 776.49 nm; calcium: 422.67 nm; magnesium: 285.21 nm) were set by WinLab32™ for AA software (PerkinElmer^®^ Inc., Waltham, MA, USA).

### Determination of amylase activity

Amylase activity was assayed via UV/VIS spectrophotometer following the method of Bernfield (1955). For enzyme extraction, 500 mg fresh samples were homogenized in eight mL deionized water at room temperature. The mixture was centrifuged at 8,000×*g* rpm for 20 min at 4 °C. The supernatant was used for amylase assay at 540 nm. The reaction system included one mL of 1% starch solution (one g soluble starch dissolved in 100 mL 0.1-mol/L citrate buffer pH 5.6) and one mL of 10 times diluted supernatant and was incubated at 40 °C for 10 min. 0.5 mL of two-mol/L NaOH was then added to stop the enzymatic reaction. Reducing sugar was then determined by the DNS colorimetric method (Li, 2006). The enzyme activity was expressed as mg sugar released per min per mg protein. The soluble protein content of the extracted supernatant was measured following the method of Bradford (1976).

### Determination of protease activity

The protease assay was performed via UV/VIS spectrophotometer as described by Zhang et al. (2017). A total of 500 mg fresh samples were homogenized with five mL 0.02 mol/L PBS (pH 7.5) and then centrifuged at 8,000×*g* rpm for 20 min at 4 °C. Enzyme activity was then measured following the method of McDonald & Chen (1965) and expressed as mg peptides decomposed by the protease from 2% casein solution (two g casein in 100 mL 0.02 mol/L PBS pH 7.5) per min per mg protein at 660 nm. Tyrosine was used as the standard for quantification by the Folin colorimetric method (Lowry et al., 1951).

### Determination of oxidation pathway related enzyme activities

For malate dehydrogenase (MDH), phosphoglucose isomerase (PGI), and glucose 6-phosphate dehydrogenase (G6PDH) assays, 500 mg fresh samples were homogenized with five mL 0.1 mmol/L Tris–HCl buffer (pH 7.4) that contained one mmol/L EDTA-Na_2_ and 1.5-mmol/L AsA and then centrifuged at 12,000×*g* rpm for 50 min at 4 °C. MDH activity was determined with assay kit (Nanjing Jiancheng Bioengineering Institute, Jiangsu, China) by referring to Bergmeyer (1956), while PGI and G6PDH activities were measured via UV/VIS spectrophotometer at 520 and 340 nm, respectively, by procedures provided by Brown & Wray (1968) and Simcox & Dennis (1978).

To measure peroxidase (POD) and polyphenoloxidase (PPO) activities of the pericarp and the seed coat via UV/VIS spectrophotometer, 500 mg fresh samples were ground and homogenized in a pre-chilled mortar with five mL of 100 mmol/L PBS (pH 6.0) that included one mmol/L EDTA-Na_2_ and 1.5-mmol/L AsA. The supernatant was recovered by centrifugation (10,000×*g* rpm for 20 min at 4 °C). POD activity was measured after the method of Kochba, Lavee & Spiegel-Roy (1977) and Feng et al. (2017). The reaction system included 0.1 mL enzyme extract and 2.9 mL 50 mmol/L PBS (pH 5.5), 1.0 mL 2% H_2_O_2_, and 1.0 mL 50 mmol/L guaiacol. POD activity was expressed kinetically, whereby the absorbance increase at 470 nm due to the oxidation of guaiacol that was detected spectrophotometrically. PPO activity was measured by monitoring oxidation of 1,2-dihydroxybenzene at 420 nm (Hao, Cang & Xu, 2004). The reaction mixture (30 °C) consisted of 0.5 mL enzyme extract, 3.5 mL 50 mmol/L PBS (pH 6.0), and 1.0 mL 100 mmol/L 1,2-dihydroxybenzene.

For pericarp and seed coat superoxide dismutase (SOD) activity measurement via UV/VIS spectrophotometer, 500 mg fresh samples were homogenized with five mL 50 mmol/L PBS (pH 7.8) that contained one mmol/L EDTA-Na_2_ and 1.5 mmol/L AsA and then centrifuged at 10,000×*g* rpm for 20 min at 4 °C. The supernatant was used for the quantification of SOD activity at 560 nm via reaction system provided by Li (2006).

The malondildehyde (MDA) content was measured via UV/VIS spectrophotometer by the thiobarbituric acid method (Janero, 1990). A total of 500 mg fresh samples were homogenized with five mL 5% trichloroacetic acid and then centrifuged at 3,000×*g* rpm for 20 min at 15 °C. Absorbance at 450, 532, and 600 nm of the supernatant was measured with a spectrophotometer to conduct the MDA calculation.

### Statistics analysis

Values were expressed as mean ± SD of independent experiments. Excel (Office 2018 Pro Plus; Microsoft Corporation, Redmond, WA, USA) was used to process figures. One-way analysis of variance with repeated measures, Duncan’s multiple range testing, and calculation of Spearman’s rank correlation coefficients were performed using SigmaPlot 14.0 (Systat Software Inc., San Jose, CA, USA). *P*-values < 0.05 were considered significant within groups. Relationships between mineral contents and nutrient variables in kernels were determined using Pearson’s correlation analysis.

## Results

### Change of fruit morphology

During *S. tonkinensis* fruit development, the construction of the continuum of the pericarp, seed coat, and kernel was observed (Fig. S1). The *V*_*f*_ and *S*_*f*_ raised by 177.1% and 94.2% between 30 and 110 DAF, respectively. After that, they changed slowly and reached the maximum values of 839.0 mm^3^ and 440.9 mm^2^ (Table S1). Between 30 and 70 DAF, the size of the *S. tonkinensis* seed enlarged considerably. Thereafter, the *V*_*s*_ changed stably between 133.9 and 157.3 mm^3^. The *S*_*s*_ ranged from 128.4 to 143.2 mm^2^ (Table S1). There was a significant positive correlation between fruit volume and seed volume (ρ = 0.915, *p* = 0.00003).

The FM trends of different parts of *S. tonkinensis* fruit showed different patterns. The pericarp and seed coat FM per fruit peaked at 100 DAF (613.5 mg) and 70 DAF (162.7 mg), respectively (Table 1). Kernel FM per fruit increased rapidly after 70 DAF and reached 93.9 mg at 140 DAF. Between 30 and 60 DAF, the pericarp FM ratio decreased by 8.31%, while the seed coat FM ratio increased. The pericarp FM ratio started as 79.92%; after 70 DAF, it remained stable at about 75.3% and the FM deposition center transferred to the kernel, which increased continuously (Fig. S2A).

**Table 1:** Dynamics of fresh matter, dry matter, and water content in the pericarp, seed coat, and kernel per fruit.

		Days after flowering											
		30	40	50	60	70	80	90	100	110	120	130	140
Fresh matter (mg)	Pericarp	196.2 ± 7.49^f^	306.0 ± 16.0^e^	363.4 ± 24.6^d^	341.9 ± 5.74^d^	464.9 ± 23.2^c^	537.9 ± 24.9^b^	545.4 ± 25.6^b^	613.5 ± 17.7^a^	551.7 ± 35.3^b^	567.0 ± 6.51^b^	547.1 ± 25.4^b^	468.1 ± 15.7^c^
	Seed coat	55.0 ± 7.39^e^	101.4 ± 15.7^c^	149.6 ± 23.73^ab^	143.4 ± 4.15^b^	162.7 ± 21.8^a^	141.7 ± 22.4^b^	103.9 ± 22.6^c^	105.0 ± 16.5^c^	102.1 ± 31.9^c^	81.3 ± 5.54^d^	77.5 ± 23.1^d^	73.7 ± 12.1^de^
	Kernel	0.59 ± 0.10^j^	2.93 ± 0.26^hi^	3.69 ± 0.91^gh^	5.89 ± 1.59^g^	8.88 ± 1.37^f^	28.6 ± 2.55^e^	49.5 ± 2.96^d^	65.5 ± 1.26^c^	65.6 ± 3.38^c^	76.7 ± 0.97^b^	79.6 ± 2.27^b^	93.9 ± 3.57^a^
Dry matter (mg)	Pericarp	52.7 ± 1.85^h^	75.9 ± 4.25^g^	97.2 ± 5.03^f^	110.1 ± 7.73^e^	149.3 ± 6.51^d^	156.1 ± 7.48^cd^	165.0 ± 7.74^bc^	170.4 ± 18.4^b^	168.6 ± 8.98^bc^	165.9 ± 2.24^bc^	166.2 ± 5.79^bc^	182.2 ± 5.41^a^
	Seed coat	8.41 ± 0.66^g^	15.8 ± 1.14^f^	29.2 ± 2.06^e^	40.8 ± 2.02^d^	69.5 ± 2.98^a^	69.1 ± 2.44^a^	59.0 ± 3.12^b^	61.4 ± 1.62^b^	62.5 ± 1.62^b^	54.4 ± 0.61^c^	53.0 ± 2.45^c^	59.3 ± 1.70^b^
	Kernel	0.09 ± 0.02^i^	0.46 ± 0.04^i^	0.72 ± 0.31^i^	1.68 ± 0.72^i^	3.98 ± 1.28^h^	15.5 ± 1.90^g^	27.7 ± 1.34^f^	40.0 ± 0.69^e^	47.8 ± 2.12^d^	55.0 ± 1.69^c^	59.0 ± 2.48^b^	68.6 ± 2.16^a^
Water content (%)	Pericarp	73.1 ± 0.34^b^	75.2 ± 0.68^a^	73.2 ± 0.67^b^	67.8 ± 1.86^e^	67.9 ± 0.22^e^	71.0 ± 0.11^cd^	69.8 ± 0.25^de^	72.2 ± 2.58^bc^	69.4 ± 2.43^de^	70.7 ± 0.17^cd^	69.6 ± 0.53^de^	61.1 ± 1.24^f^
	Seed coat	84.7 ± 0.20^a^	84.5 ± 0.24^a^	80.5 ± 0.65^a^	71.5 ± 0.07^b^	56.2 ± 1.34^c^	51.3 ± 0.53^d^	43.2 ± 0.50^e^	41.5 ± 2.42^e^	38.8 ± 0.34^e^	33.1 ± 2.37^f^	31.7 ± 2.12^f^	19.6 ± 2.78^g^
	Kernel	84.7 ± 0.14^a^	84.5 ± 0.44^a^	80.5 ± 1.32^b^	71.5 ± 1.93^c^	55.2 ± 1.55^d^	46.0 ± 0.64^e^	44.1 ± 0.76^e^	38.9 ± 0.16^f^	27.2 ± 2.09^g^	28.3 ± 2.19^g^	25.9 ± 1.60^g^	26.9 ± 1.53^g^

**Note:**

Different letters within days after flowering indicate significant variables difference in the pericarp, seed coat, or kernel.

Similarly, the trends exhibited by the DM varied according to the part of the fruit in *S. tonkinensis* under consideration. The accumulation of pericarp and seed coat DM increased rapidly before 70 DAF. Thereafter, the pericarp DM maintained the trend of a slow rise to 182.2 mg at 140 DAF while the seed coat DM reduced to 59.3 mg (Table 1). Kernel DM changed slowly between 30 and 70 DAF, followed by a continuous and rapid increase. The DM composition of the three parts changed more obviously than did the FM composition (Fig. S2B). The pericarp DM ratio decreased by 25% during fruit development. The DM deposition center transferred to the kernel after 70 DAF. In the end, pericarp, seed coat, and kernel DM accounted for 22.1%, 19.1%, and 58.8%, respectively.

The water content within the pericarp ranged between 67.8% and 75.2% of mass from 30 to 130 DAF and then fell to 61.1% (Table 1). The water content of the seed coat and the kernel shared the same downward trend from 30 to 100 DAF. After 100 DAF, however, the water content of the seed coat reduced continually to 19.6% while the kernel’s water content remained stable around 27.2%.

### Change of carbohydrate content

Total soluble sugar content per pericarp increased in waves during fruit development (Table 2). It reached the first peak at 70 DAF (26.6 mg) and then decreased by 24.4% (to 20.1 mg) at 80 DAF before increasing again with the same speed as the former period. Thereafter, it reached the second peak at 130 DAF (41.7 mg), ultimately dropping to 36.0 mg. The total soluble sugar content per seed coat showed an up-down pattern peaking at 80 DAF (10.1 mg). What is more, it dropped to zero after 110 DAF. Between 30 and 70 DAF, the total soluble sugar content per kernel increased slowly. A dramatic rise was then observed. At 100 DAF, a 10-day lag phase appeared before the content increased again, albeit more slowly than in the former period. Total soluble sugar concentrations based on FM during *S. tonkinensis* fruit development were shown in Table S2. The evolution of the total soluble sugar ratio among the three parts was close to the total soluble sugar content in different parts (Fig. S2C). Between 30 and 70 DAF, most sugar was distributed to the pericarp and the seed coat. Accumulation of sugar in the kernel increased thereafter. Ultimately the total soluble sugar content of the pericarp, seed coat, and kernel accounted for 74.5%, 0.66%, and 24.8%, respectively.

**Table 2:** Dynamics of nutritive contents in the pericarp, seed coat, and kernel per fruit.

		Days after flowering											
		30	40	50	60	70	80	90	100	110	120	130	140
Total soluble sugar (mg)	Pericarp	8.85 ± 0.40^h^	13.7 ± 1.60^gh^	19.6 ± 1.05^g^	18.7 ± 0.98^g^	26.6 ± 0.93^ef^	20.1 ± 2.99^fg^	28.7 ± 1.04^e^	29.8 ± 5.02^de^	32.4 ± 2.16^cd^e	39.2 ± 3.93^bc^	41.7 ± 5.29^a^	36.0 ± 1.70^bcd^
	Seed coat	3.42 ± 0.06^d^	5.64 ± 0.35^c^	7.81 ± 0.19^b^	8.37 ± 0.57^b^	8.53 ± 0.61^b^	10.1 ± 1.12^a^	3.45 ± 0.18^d^	1.71 ± 0.01^e^	1.13 ± 0.03^ef^	0.92 ± 0.06^ef^	0.85 ± 0.06^ef^	0.32 ± 0.01^f^
	Kernel	0.07 ± 0.07^k^	0.36 ± 0.25^jk^	0.70 ± 0.80^j^	1.20 ± 0.79^i^	1.80 ± 1.22^h^	4.53 ± 1.69^g^	6.18 ± 0.93^f^	7.49 ± 0.33^e^	7.09 ± 0.47^d^	8.63 ± 0.15^c^	9.54 ± 0.11^b^	12.0 ± 0.16^a^
Total starch (mg)	Pericarp	4.88 ± 0.17^f^	6.09 ± 0.50^f^	7.48 ± 0.80^ef^	10.2 ± 1.03^cde^	16.6 ± 3.13^a^	13.2 ± 0.50^bc^	12.2 ± 2.36^bcd^	10.8 ± 1.17^cd^e	10.3 ± 1.13^cde^	9.52 ± 2.79^de^	14.4 ± 2.19^ab^	14.9 ± 2.53^ab^
	Seed coat	0.48 ± 0.05^d^	0.60 ± 0.07^d^	1.03 ± 0.06^cd^	1.70 ± 0.30^bc^	5.91 ± 0.88^a^	5.09 ± 0.92^a^	2.41 ± 0.29^b^	2.70 ± 0.36^b^	2.33 ± 0.13^b^	2.05 ± 0.21^b^	2.02 ± 0.27^b^	2.33 ± 0.22^b^
	Kernel	0.002 ± 0.00^e^	0.008 ± 0.00^e^	0.009 ± 0.00^e^	0.009 ± 0.002^e^	0.013 ± 0.00^e^	0.07 ± 0.00^e^	0.25 ± 0.05^d^	0.41 ± 0.04^d^	0.65 ± 0.06^c^	1.09 ± 0.09^b^	1.11 ± 0.12^b^	1.75 ± 0.15^a^
Total free amino acid (μg)	Pericarp	15.2 ± 3.49^h^	20.0 ± 0.33^fgh^	27.9 ± 6.55^ef^h	51.8 ± 7.19^bc^	45.1 ± 1.20^cd^	48.4 ± 6.53^bcd^	63.4 ± 11.9^a^	58.0 ± 11.6^ab^	29.9 ± 1.30^ef^	38.3 ± 0.85^de^	31.5 ± 8.16^ef^	16.8 ± 1.27^gh^
	Seed coat	2.99 ± 0.47^d^	6.19 ± 2.01^cd^	8.68 ± 1.25^bc^	12.8 ± 3.06^b^	17.9 ± 7.98^a^	20.9 ± 2.99^a^	6.20 ± 0.05^cd^	2.58 ± 0.20^d^	5.85 ± 0.09^cd^	3.14 ± 0.73^d^	3.93 ± 1.08^cd^	2.53 ± 0.56^d^
	Kernel	0.06 ± 0.00^c^	0.33 ± 0.06^c^	0.40 ± 0.01^c^	0.96 ± 0.11^c^	2.03 ± 0.09^c^	9.11 ± 1.07^b^	8.01 ± 0.65^b^	11.7 ± 1.71^a^	12.2 ± 1.41^a^	7.96 ± 1.00^b^	8.78 ± 1.16^b^	11.2 ± 2.31^a^
Total soluble protein (mg)	Pericarp	0.24 ± 0.01^f^	0.37 ± 0.04^e^	0.44 ± 0.02^e^	0.43 ± 0.02^e^	0.63 ± 0.02^cd^	0.74 ± 0.06^b^	0.63 ± 0.01^cd^	0.71 ± 0.04^b^	0.70 ± 0.05^bc^	0.74 ± 0.03^b^	0.82 ± 0.07^a^	0.60 ± 0.07^d^
	Seed coat	2.86 ± 0.09^e^	3.94 ± 0.62^d^	6.37 ± 0.40^b^	5.44 ± 0.54^c^	8.49 ± 0.71^a^	8.77 ± 1.26^a^	1.81 ± 0.24^f^	0.95 ± 0.05^g^	0.20 ± 0.01^g^	0.14 ± 0.01^g^	0.12 ± 0.01^g^	0.10 ± 0.00^g^
	Kernel	0.03 ± 0.00^e^	0.16 ± 0.01^e^	0.26 ± 0.05^e^	0.46 ± 0.04^e^	0.82 ± 0.07^de^	2.81 ± 0.05^d^	6.36 ± 0.05^c^	8.45 ± 0.98^c^	8.70 ± 1.03^c^	13.0 ± 1.45^b^	14.3 ± 1.38^ab^	16.8 ± 1.78^a^
Total oil* (mg)	Kernel	0.00 ± 0.00	0.00 ± 0.00	0.01 ± 0.00	0.08 ± 0.01	1.42 ± 0.12	8.59 ± 0.15	13.86 ± 0.48	18.31 ± 0.45	21.03 ± 0.95	23.53 ± 0.85	29.79 ± 1.48	38.42 ± 0.93

**Notes:**

Different letters within days after flowering indicate significant variables difference in the pericarp, seed coat, or kernel.

* Total oil content of each kernel sample was cited from our published paper (Zhang et al., 2017).

The total starch content of the pericarp and the seed coat per fruit shared a similar upward trend (ρ = 0.786, *p* = 0.00244), reaching a peak of 16.5 and 5.91 mg at 70 DAF, respectively (Table 2). After that, the former showed a 50-day decrease and 20-day increase trend, while the latter showed a 20-day decrease trend and then remained steady. The total starch of the kernel appeared and began to accumulate at 70 DAF. The total amount of starch in the kernel remained low. Total starch concentrations based on FM during *S. tonkinensis* fruit development were shown in Table S2. According to the starch ratio among the three fruit parts, the predominant starch deposition center switched from the seed coat to the kernel after 80 DAF. The amount of starch in the pericarp remained largely constant during fruit development (Fig. S2D). In the end, the total starch content of the pericarp, seed coat, and kernel accounted for 78.5%, 12.3%, and 9.2%, respectively.

**Figure 1:**
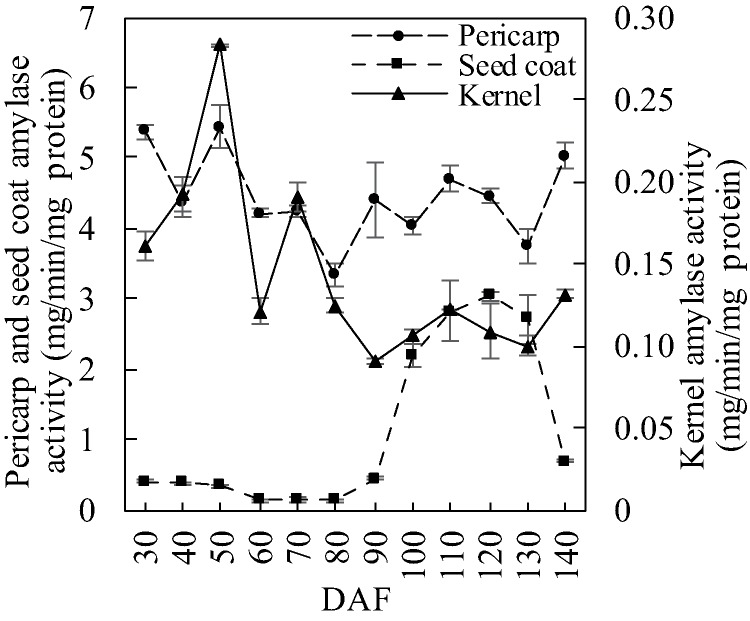
Amylase activity dynamics in developing *S. tonkinensis* fruit.

Pericarp amylase activity showed a fluctuating trend during *S. tonkinensis* fruit development with a valley value at 80 DAF (3.35 mg/min/mg protein) (Fig. 1). The seed coat amylase activity appeared hyperbolic, exhibiting a 50-day slow downward trend and then a rapid increase between 80 and 120 DAF before a 20-day precipitous fall. During the last 20 days, the seed coat amylase activity dropped from 3.03 to 0.69 mg/min/mg protein. Amylase activity in the kernel and in the pericarp both showed wavelike trends, whereas kernel amylase activity stayed fairly constant at a low level.

**Figure 2:**
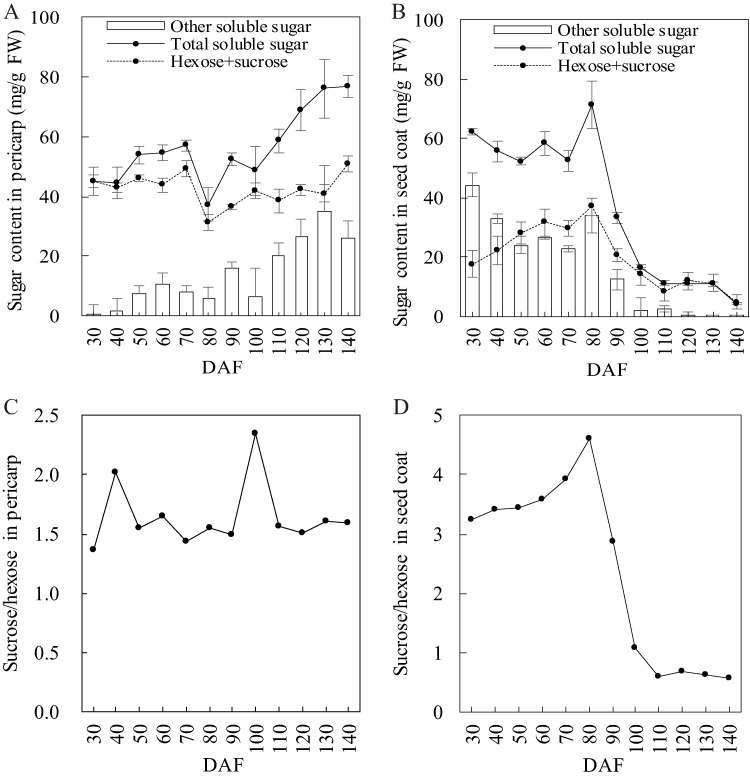
Sugar composition evolution in developing *S. tonkinensis* pericarp and seed coat. (A) Total soluble sugar, hexose + sucrose and other soluble concentration in pericarp; (B) Total soluble sugar, hexose + sucrose and other soluble concentration in seed coat; (C) Evolution of sucrose/hexose ratio in pericarp; (D) Evolution of sucrose/hexose ratio in seed coat.

The evolution of the hexose and sucrose concentration in the developing pericarp mirrored the same pattern as total soluble sugar, having the lowest amount present at 80 DAF (Fig. 2A). The total soluble sugar was mainly composed of fructose, glucose, and sucrose at 30 DAF. After 30 DAF, other soluble sugar appeared and increased continuously in the pericarp, peaking at 130 DAF. Conversely, other soluble sugar in the seed coat decreased, especially at 90 DAF, and then dropped to zero (Fig. 2B). The evolution of the glucose, fructose, and sucrose concentration in the developing seed coat was different than that of the total soluble sugar, but they both peaked at 80 DAF. In addition, the evolution of the sucrose to hexose (glucose and fructose) ratio in the pericarp stayed steady at a stable level except for two peaks at 40 and 100 DAF (Fig. 2C). This was different than the respective trend exhibited by the seed coat (Fig. 2D).

### Change of amino acid and soluble protein content

The total free amino acid content of the pericarp per fruit had a large portion and showed an up-down pattern (Table 2) with three peaks at 60 DAF (51.8 μg), 90 DAF (63.5 μg), and 120 DAF (38.3 μg), respectively. After that, it decreased continuously and approximately reached the initial level (16.8 μg) at 140 DAF. The total free amino acid content of the seed coat had a continuous increase between 30 and 80 DAF that was followed by a 10-day dramatic drop (70.4%). Thereafter, the content remained stable. Total free amino acid concentrations based on FM during *S. tonkinensis* fruit development were shown in Table S2. Amino acids began to accumulate in the kernel at 60 DAF and then gradually increased to a peak (12.2 μg) at 110 DAF. The change of the free amino acid ratio of the three fruit parts followed no obvious rule (Fig. S2E). Ultimately, the total free amino acid contents of the pericarp, seed coat, and kernel accounted for 55.0%, 8.3%, and 36.7%, respectively.

The total soluble protein content in the pericarp showed an upward trend during fruit development, although the content remained below one mg in any given pericarp (Table 2). The total soluble protein content of the seed coat had a similar trend to that of the total free amino acid, exhibiting a peak at 80 DAF (8.77 mg) and then dropping sharply to zero. The total soluble protein content of the kernel increased slowly between 30 and 70 DAF. A dramatic change was then observed after 70 DAF. A 10-day lag phase appeared at 100 DAF. This was followed by another increase that occurred with the same speed as the former period. Total soluble protein concentrations based on FM during *S. tonkinensis* fruit development were shown in Table S2. According to the soluble protein ratio among the different parts of the fruit, the primary locus of the protein deposition center was in the seed but not in the pericarp (Fig. S2F). Furthermore, the seed coat began to provide a nitrogen source to the kernel between 80 and 90 DAF. In the end, the total soluble protein content of the pericarp, seed coat, and kernel accounted for 3.45%, 0.57%, and 95.98%, respectively.

**Figure 3:**
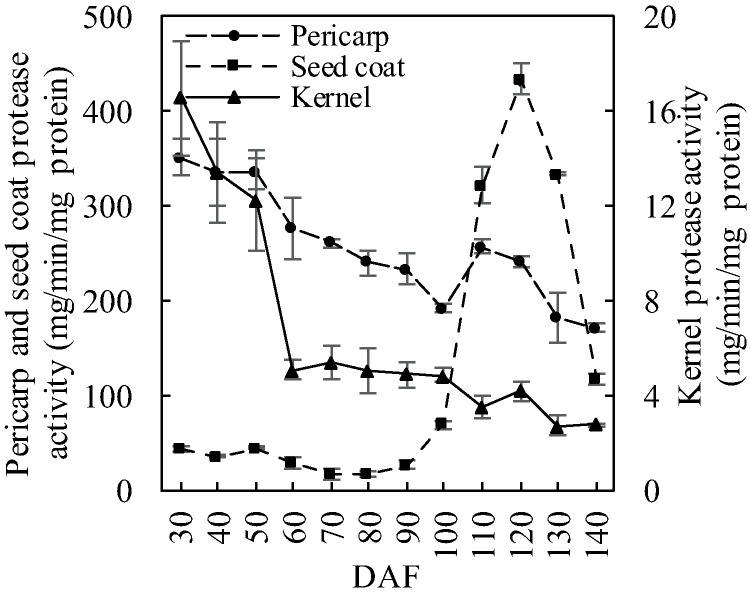
Protease activity dynamics in developing *S. tonkinensis* fruit.

Pericarp protease activity stayed high at previous 20 days and then dropped to the minimum value (192.9 μg/min/mg protein) at 100 DAF (Fig. 3). It dropped again between 110 and 140 DAF with the same speed of decline of the former period. The trend of the change in seed coat protease activity was close to that of the amylase activity (ρ = 0.908, *p* = 0.0000451), peaking at 120 DAF (433.6 μg/min/mg protein). Kernel protease activity showed a gradual downward trend with a sharp decrease (58.6%) between 50 and 60 DAF. It then remained stable until maturation.

### Change of chlorophyll content

**Figure 4:**
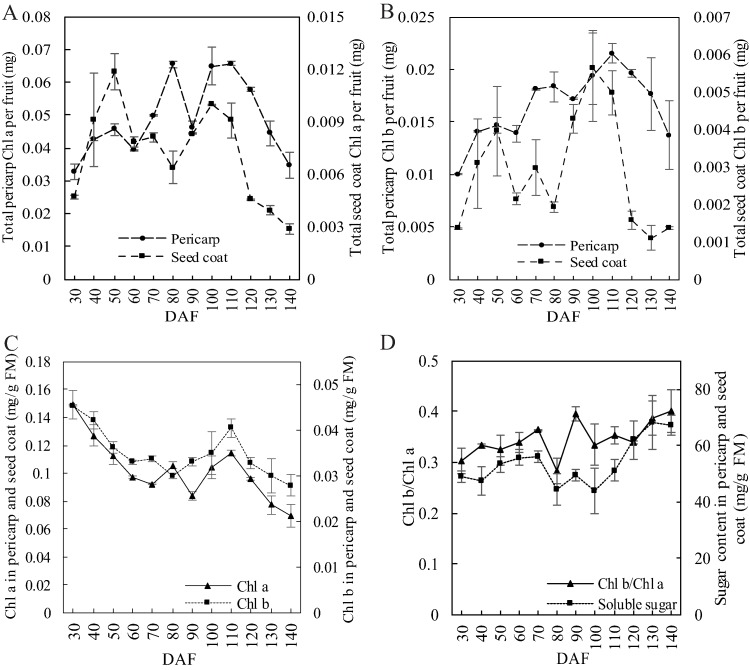
Chlorophyll content evolution in developing *S. tonkinensis* pericarp and seed coat. (A) Total Chl a content in pericarp and seed coat per fruit; (B) Total Chl b content in pericarp and seed coat per fruit; (C) Concentration of Chl a and b in the combined parts of pericarp and seed coat; (D) Concentration of soluble sugar and Chl b/a in the combined parts of pericarp and seed coat.

Total Chl a content in the pericarp maintained a 50-day increase and reached 0.065 mg at 80 DAF. It then dropped to 0.046 mg before again rising to 0.065 mg. It dropped sharply to its initial level (0.035 mg) during the last 30 days (Fig. 4A). Total Chl a content in the seed coat was obviously lower than in the pericarp, with two peaks at 50 DAF (0.012 mg) and 100 DAF (0.010 mg), respectively. The change in the Chl b content of the pericarp or the seed coat was different than that of Chl a (Fig. 4B). The total Chl b content of the pericarp showed stepped increase and reached a peak (0.022 mg) at 110 DAF, thereafter it went down synchronously with the pericarp Chl a concentration. The trend of the change in the Chl b in the seed coat also had two peaks at 50 DAF (0.0040 mg) and 100 DAF (0.0056 mg), one large and one small, which were opposite to Chl a. Overall, the total chlorophyll concentration in fruit FM showed a downward trend, although the Chl a and Chl b content increased in the late stage (Fig. 4C). Furthermore, the change in the ratio of Chl b/a had a significant correlation (ρ = 0.652, *p* = 0.0217) with the trend of the total sugar content in the pericarp and the seed coat (Fig. 4D).

### Changes of minerals

The potassium content in the pericarp had an overall upward trend during fruit development with a higher value than that of the seed coat and the kernel at each time (Table 3). It increased slowly during previous 30 days and peaked at 70 DAF (0.97 mg). A 10-day decrease followed before the content increased again with the same speed as during the former period. The potassium content of the pericarp peaked again at 120 DAF (1.68 mg) and then decreased to 1.52 mg, which was 692.8% higher than the original content. The potassium content of the seed coat presented an earlier increase than that of the pericarp, followed by a downward trend, peaking at 50 DAF (0.19 mg). Total potassium concentrations based on FM during *S. tonkinensis* fruit development were shown in Table S2. The potassium content of the kernel appeared and began to accumulate at 60 DAF with a 20-day lag phase between 100 and 120 DAF. It increased to 0.25 mg ultimately. The potassium deposition center transferred to the kernel after 70 DAF. In the end, the pericarp, seed coat, and kernel potassium content accounted for 84.72%, 1.54%, and 13.74%, respectively (Fig. S2G).

**Table 3:** Dynamics of mineral contents in the pericarp, seed coat, and kernel per fruit.

		Days after flowering											
		30	40	50	60	70	80	90	100	110	120	130	140
Potassium (mg)	Pericarp	0.19 ± 0.00^h^	0.30 ± 0.02^gh^	0.41 ± 0.02^fg^	0.45 ± 0.04^f^	0.97 ± 0.03^d^	0.82 ± 0.02^e^	0.90 ± 0.08^de^	1.38 ± 0.13^c^	1.48 ± 0.07^bc^	1.68 ± 0.05^a^	1.49 ± 0.08^bc^	1.53 ± 0.15^b^
	Seed coat	0.05 ± 0.00^g^	0.10 ± 0.00^f^	0.19 ± 0.02^a^	0.15 ± 0.00^bc^	0.16 ± 0.01^b^	0.12 ± 0.00^de^	0.12 ± 0.01^de^	0.13 ± 0.00^cd^	0.11 ± 0.01^ef^	0.06 ± 0.00^g^	0.04 ± 0.00^h^	0.03 ± 0.01^h^
	Kernel	0.000 ± 0.00^f^	0.001 ± 0.00^f^	0.001 ± 0.00^f^	0.001 ± 0.00^f^	0.02 ± 0.00^f^	0.05 ± 0.00^e^	0.08 ± 0.01^d^	0.10 ± 0.00^c^	0.11 ± 0.01^c^	0.11 ± 0.00^c^	0.13 ± 0.01^b^	0.25 ± 0.02^a^
Calcium (mg)	Pericarp	1.76 ± 0.05^ef^	3.49 ± 1.07^e^	6.53 ± 1.96^d^	6.23 ± 2.10^d^	10.8 ± 0.11^bc^	0.25 ± 0.02^f^	0.26 ± 0.02^f^	8.25 ± 3.11^cd^	10.4 ± 2.14^c^	14.3 ± 0.12^a^	13.4 ± 1.70^ab^	8.69 ± 2.16^cd^
	Seed coat	0.25 ± 0.02^d^	1.73 ± 0.02^b^	2.13 ± 0.43^a^	2.39 ± 0.14^a^	2.40 ± 0.06^a^	1.51 ± 0.15^bc^	1.34 ± 0.35^c^	0.43 ± 0.07^d^	0.18 ± 0.00^d^	0.35 ± 0.02^d^	0.13 ± 0.01^d^	0.16 ± 0.08^d^
	Kernel	0.00 ± 0.00^g^	0.00 ± 0.00^g^	0.03 ± 0.01^g^	0.06 ± 0.00^g^	0.21 ± 0.03^f^	0.21 ± 0.01^f^	0.37 ± 0.05^e^	0.56 ± 0.02^d^	0.61 ± 0.08^cd^	0.67 ± 0.04^c^	0.77 ± 0.10^b^	0.90 ± 0.06^a^
Magnesium (mg)	Pericarp	0.06 ± 0.00^ef^	0.11 ± 0.02^cd^	0.08 ± 0.02^def^	0.08 ± 0.01^de^	0.10 ± 0.02^cd^	0.05 ± 0.01^f^	0.06 ± 0.00^ef^	0.15 ± 0.03^ab^	0.14 ± 0.00^abc^	0.17 ± 0.03^a^	0.15 ± 0.01^ab^	0.13 ± 0.03^bc^
	Seed coat	0.01 ± 0.00^g^	0.03 ± 0.00^e^	0.05 ± 0.00^c^	0.06 ± 0.00^b^	0.07 ± 0.00^a^	0.05 ± 0.00^c^	0.04 ± 0.00^d^	0.04 ± 0.00^d^	0.03 ± 0.00^e^	0.02 ± 0.00^f^	0.01 ± 0.00^g^	0.03 ± 0.00^e^
	Kernel	0.00 ± 0.00^f^	0.00 ± 0.00^f^	0.00 ± 0.00^f^	0.00 ± 0.00^f^	0.01 ± 0.00^f^	0.02 ± 0.00^e^	0.05 ± 0.01^d^	0.07 ± 0.00^c^	0.07 ± 0.01^c^	0.07 ± 0.00^c^	0.08 ± 0.00^b^	0.10 ± 0.01^a^

**Note:**

Different letters within days after flowering indicate significant variables difference in the pericarp, seed coat, or kernel.

The calcium content of the pericarp showed an M-shaped trend (Table 3). The content increased between 30 and 70 DAF and reached the first peak at 70 DAF (10.8 mg). There was very little calcium present in the pericarp between 80 and 90 DAF. Thereafter the content increased again and reached the second peak at 120 DAF (14.3 mg). The calcium content of the seed coat increased to 2.40 mg at 70 DAF and then decreased to about 0.20 mg after 100 DAF. The calcium content continuously increased, ultimately reaching 0.90 mg at last. Total calcium concentrations based on FM during *S. tonkinensis* fruit development were shown in Table S2. The calcium deposition center varied at different points in time. Between 30 and 70 DAF, the pericarp and the seed coat were the main deposition center for calcium. Then, between 80 and 90 DAF, the seed coat and the kernel became the main deposition center. Thereafter the pericarp became the main deposition center again and accounted for about 89.15% in the end, while the seed coat and the kernel accounted for 1.64% and 9.21%, respectively (Fig. S2H).

The magnesium content of the pericarp showed a double-M-shaped trend with stepwise accumulation (Table 3). The first M-shaped pattern was present between 30 and 80 DAF with two peaks at 40 DAF (0.11 mg) and 70 DAF (0.11 mg), respectively. The second M-shaped pattern was present from 90 to 140 DAF with two peaks at 100 DAF (0.15 mg) and 130 DAF (0.17 mg), respectively. The magnesium content of the seed coat showed an upward and then downward trend, peaking at 70 DAF (0.07 mg), while that in the kernel showed a similar S-shaped trend with rapid accumulation between 70 and 100 DAF. Total magnesium concentrations based on FM during *S. tonkinensis* fruit development were shown in Table S2. Across the continuum, the magnesium deposition center was in the seed coat from 30 to 80 DAF and then in the kernel thereafter. In the end, the pericarp, seed coat, and kernel magnesium content accounted for 50.94%, 10.56%, and 38.50%, respectively (Fig. S2I).

### Changes of oxidation pathways

During fruit development, three enzymes related to the glycoxidation pathway varied with two peaks (Table S3). PGI and MDA reached the first peak at 70 DAF (912.1 and 0.74 U/min/mg protein). After a 10-day decrease, they increased again and reached the second peak at 120 DAF (930.3 and 0.67 U/min/mg protein). G6PDH activity showed two peaks at 60 DAF (20.2 U/min/mg protein) and 100 DAF (21.6 U/min/mg protein), respectively. It maintained a slow downward trend during the last 40 days. The evolution of the three enzyme activities in the seed coat was different to these of the pericarp. PGI activity showed an M-shaped trend that peaked at 50 DAF (99.7 U/min/mg protein) and 130 DAF (248.1 U/min/mg protein). MDH activity remained stable with a little downward trend except at 90 DAF (0.43 U/min/mg protein). G6PDH activity varied around 40 U/min/mg protein between 30 and 80 DAF and then showed a gradual decrease to none at 140 DAF.

Polyphenoloxidase activity in the pericarp was higher than that in the seed coat during fruit development overall (Table S3). A similar result was shown in POD activity dynamics. Pericarp PPO activity showed a wavelike rise with two peaks at 80 DAF (27.3 U/min/g FW) and 120 DAF (33.7 U/min/g FW). Seed coat PPO activity kept increase gradually between 30 and 100 DAF. Following a 10-day rapid decrease by 71.7%, it maintained steadily at about 4.5 U/min/g FW. Compared to its initial stage, the final pericarp PPO activity increased by 30%. Seed coat PPO activity remained unchanged. Pericarp POD activity showed a wide W-shaped trend with a peak (1.95 U/min/g FW) at 80 DAF. Seed coat POD activity peaked (0.78 U/min/g FW) at 70 DAF and sharply dropped to its initial level. Thereafter it remained unchanged but increased slightly at 130 DAF.

Superoxide dismutase activity in the pericarp increased between 30 and 50 DAF and remained stable around 1,300 U/min/g FW thereafter (Table S3). SOD activity in the seed coat maintained a low value during the previous 50 days and increased rapidly between 80 and 90 DAF. After that, it remained stable around 550 U/min/g FW. There was a significant positive correlation between the MDA content in the pericarp and in the seed coat (ρ = 0.842, *p* = 0.000587), both of which showed waves trending downward during the whole experiment (Table S3).

## Discussion

### Morphology change varies in *S. tonkinensis* fruit and seed

***Styrax* tonkinensis** seeds grew rapidly and reached the maximum size in a short time (from 30 to 70 DAF), sharing a similar dynamic growth tendency with *Prunus sibirica* and *Lophantera lactescens*, which are widely used for biodiesel and ornamental planting (Wang et al., 2019; Silva et al., 2019). The volume and surface area of the seed remained stable after 70 DAF, whereas that of the fruit increased continually (Table S1). The change in the water content of the seed, which remained high initially and then reduced to a hygroscopic balance (Table 1), facilitates transportation and accumulation during seed development (Matheus, Lopes & Correa, 2011). Pericarp water content, which remained high during *S. tonkinensis* fruit development, may be related to continuous photosynthesis and DM accumulation (Zhang et al., 2018a). During fruit maturation, there was a rapid reduction in pericarp water content. Then, the fruit splits of its own accord along the carpel line leading to seed shedding (Silva et al., 2017). A relevant study on the molecular mechanism of silique dehiscence in *Arabidopsis thaliana* has been conducted (He et al., 2018), which would inspire some further studies in *S. tonkinensis*.

### The role of the pericarp and the seed coat in fruit nutrient distribution

We analyzed the DM distribution in the continuum of the pericarp, seed coat, and kernel during *S. tonkinensis* fruit development. Overall, the accumulation and deposition center of DM in fruit was primarily in the seed coat between 30 and 70 DAF. It then transferred to the kernel. The percentage of pericarp dry weight trended downward during the whole development of the fruit. As a result, we deduced a hypothesis: the pericarp and the seed coat may comprise a nutrient buffer storage area between the mother tree and the kernel.

Firstly, soluble sugar concentration is correlated with fruit development and abscission (Mehouachu et al., 1995; Mehouachi et al., 2000). The sucrose/hexose ratio has a positive effect on deposition of storage reserves in kernels (Zhang et al., 2017). We also found that the rise of the sucrose/hexose ratio affected nutrient accumulation in the pericarp and the seed coat. The sucrose/hexose ratio in the pericarp had two peaks at 40 and 100 DAF (Fig. 2C), respectively, with accumulation peaks or acceleration of soluble sugar, starch and protein deposition in the following 20–30 days (Table 2). The ratio of sucrose/hexose in the seed coat increased between 30 and 80 DAF (Fig. 2D). During this period, three nutrient concentrations exhibited a continuous upward trend.

Secondly, 70–80 DAF is an important point in time during *S. tonkinensis* fruit development. At this point, nutrients in the pericarp and the seed coat dropped rapidly. Oil, total soluble starch, and protein content, however, started to increase in the kernel (Zhang et al., 2017). This indicates that the deposition center of stored nutrients was transferred from the pericarp or seed coat to the kernel. That said, the carbon and nitrogen sources in the kernel were not entirely came from pericarp and seed coat, with the decreased protein content in the pericarp and the seed coat below the increased content in the kernel. Even more remarkably, the total soluble sugar and the protein content in the pericarp increased again after maintaining a 10-day downward trend at 80 DAF. Meanwhile, enzyme activities related to the glycoxidation pathway, that is, PGI, MDH, and G6PDH, as well as to respiratory metabolism, that is, POD and PPO, peaked at 70 or 80 DAF (Table S3) while the pericarp and the seed coat showed improved antioxidant system activity for dealing with MDA produced by hypermetabolism. Additionally, the increase of POD activity in the pericarp and the seed coat may be related to the final maturity of the fruit. The increase of POD activity, correlating with the decrease of indoleacetic acid oxidase, contributes to the accumulation of indoleacetic acid (Zhang et al., 2017). Also, the increase of POD activity enhances biosynthesis of lignin, cork layer, and hydroxyproline glycoprotein to strengthen the stability of cell walls (Lewis & Yamamoto, 1990; Passardi et al., 2005). Therefore, the increase of POD during maturation may affect lignification of the cells of the pericarp and seed coat.

Finally, referring to previous studies (Zhang et al., 2017, 2018b), the nutrient buffer storage area in the pericarp and the seed coat may be associated with carbon competition between FAs and starch in the kernel. On the one hand, FAs synthesis rate in the kernel was high before 80 DAF, which would be decreased if the carbon and nitrogen sources were transferred into it during this period, whereas the number and size of oil bodies surrounded by oil-body-membrane protein increased continuously (Zhang et al., 2018b). On the other hand, with the continuous input of photosynthetic products from leaves, nutrients such as starch were stored temporarily in the pericarp and the seed coat. Nutrient transport into the kernel, therefore, would be taking place when FAs accumulation began to decrease (Zhang et al., 2017).

### The carbon contribution of the green pericarp and the seed coat to the fruit

The fruit color is mainly determined by the photosynthetic pigments in the pericarp, including chlorophyll, xanthophyll, and zeaxanthin (Yemis, Bakkalbasi & Artik, 2012; Wang et al., 2017). Many studies concerning chlorophyll focus on the color change of the developmental pericarp (Deylami et al., 2016; Fang et al., 2017) rather than explore the relationship between pericarp chlorophyll content and seed nutrients. The castor capsule wall possesses photosynthetic ability during seed filling and contributes significantly to carbon fixation and seed yield (Zhang, Mulpuri & Liu, 2016). Similar research has analyzed this in canola and black rice (Yan et al., 2009; Rahman, Lee & Kang, 2015), showing that fruit is not only an important storage organ but also an important photosynthetic organ as it acts as both a pool and a source. Within a certain range, there is a significant correlation between chlorophyll content and photosynthetic rate (Mae, 1997). For edible fruit, the photosynthate is mostly distributed in the fleshy pericarp (Li et al., 2009). For other fruit, however, it is mostly in the kernel, resulting in the kernel’s concentration of carbon and nitrogen was much higher than the concentration of each in the pericarp in *S. tonkinensis*. What is the carbon contribution of the green pericarp and the seed coat to the fruit? The chlorophyll concentration of the *S. tonkinensis* pericarp and seed coat remained depressed as of 30 DAF, but had a peak at a mid-later development stage (80–120 DAF) (Fig. 4C), indicating an increase in the rate of photosynthesis in both the pericarp and the seed coat. Similar results were analyzed in *Litchi chinensis* (Gao et al., 2000). The change of peaks in Chl a content in the seed coat was opposite to Chl b, which made Chl b/a value rapidly increase at a mid-late period of *S. tonkinensis* fruit development (Fig. 4D). During this period, the seed coat began to lignify and prevent the transmittance of long wavelengths light. The Chl b/a ratio increased in order to adapt to the change in the light environment, improving the utilization of high-light (Qin & Wang, 2009). At 80 DAF, oil accumulation in the kernel slowed down and FA concentration decreased, whereas starch began to accumulate. Meanwhile, nutrients began to be transported to the kernel. Overall, the ascension of photosynthesis in the green pericarp and seed coat may provide a carbon precursor for starch biosynthesis in the pericarp.

### The relationship between nutritive component and minerals

Kernel oil contents during *S. tonkinensis* fruit development in previous study (Zhang et al., 2017) were presented in Table 2. And the correlation between the concentration of three minerals and the concentration of four nutrients (sugar, starch, oil, and protein) was analyzed (Table 4). The quantity of potassium had a significant positive correlation with starch (ρ = 0.673, *p* = 0.0164), oil (ρ = 0.915, *p* = 0.000203), and protein concentration (ρ = 0.814, *p* = 0.00128), respectively. The amount of magnesium present had a significant positive correlation with starch (ρ = 0.705, *p* = 0.0104), oil (ρ = 0.913, *p* = 0.000228), and protein content (ρ = 0.896, *p* = 0.0000786), respectively. Calcium content had a significant correlation with the soluble sugar content (ρ = 0.585, *p* = 0.0457). The lay phase stage between 80 and 120 DAF of potassium content related to the existence of a period of oil accumulation speed slow down during *S. tonkinensis* seed development (Table 3) (Zhang et al., 2017).

**Table 4:** Correlation between mineral components and nutritive components in the kernel.

	Sugar	Starch	Oil	Protein
Potassium	−0.331	0.673*	0.915**	0.814**
Calcium	0.585*	−0.00845	−0.184	0.240
Magnesium	−0.526	0.705*	0.913**	0.896**

**Notes:**

* Means significant at the level of 0.05.

** Means significant at the level of 0.01.

Plants, especially fruit trees, need a large quantity of potassium for their growth. The potassium mainly exists in the metabolic pool, that is, earthnut, tuber and seed, to accelerate transportation and transformation of photosynthate and to be an activator of many enzymes (Cherel, 2004). Potassium is closely related to oil accumulation during oil seed development. For many species, Potassium fertilizer can increase the oil content (Parveen et al., 2016; Szczepaniak et al., 2017; Hu et al., 2018). The same results were verified in developmental *S. tonkinensis* seeds in our later research of Zhang et al. Magnesium, also related to the carbohydrate metabolism and lipids synthesis (Pawar et al., 2018), showed a similar tendency with potassium, having significant positive correlations with nutrient components. Calcium, having a high amount in kernel, just showed a significant correlation with the soluble sugar content. The functional relationship between minerals and nutrients is necessary to be further studied.

## Conclusions

During *S. tonkinensis* fruit development, the maternal unit, that is, the pericarp and seed coat, may act a nutrient buffer storage area between the mother tree and the kernel which is affected by the carbon competition between FAs and starch in the kernel. It is an important time point from 70 to 80 DAF for the nutrient distribution in the continuum of the pericarp, seed coat, and kernel. Around this time, the DM deposition center in the continuum is transferred from the seed coat to the kernel. Metabolic enzyme activities have reached a turning point, while the nutrients, such as oil, sugar, starch, protein, potassium, and magnesium deposition centers are transferred to the kernel from the pericarp or the seed coat successively. The Chl b/a ratio in the maternal unit has a significant positive correlation with the total soluble sugar concentration. The increase of the sucrose to hexose ratio foreshadows a rapid accumulation of nutrients. In general, the nutrient distribution in the continuum of the pericarp, seed coat, and kernel is very active and methodical during the entirety of *S. tonkinensis* fruit development, storing adequate nutrients for the next generation, that is, the biofuel seed with high oil content.

## Supplemental Information

10.7717/peerj.7996/supp-1 Supplemental Information 1 Photograph show longitudinal sections of *Styrax tonkinensis* fruit at 120 DAF (2.5×).

10.7717/peerj.7996/supp-2 Supplemental Information 2 Evolution of nutrient composition among different parts during *S. tonkinensis* fruit development. (A) Fresh matter; (B) Dry matter; (C) Soluble sugar; (D) Starch; (E) Free amino acids; (F) Soluble protein; (G) Potassium; (H) Calcium; (I) Magnesium.

10.7717/peerj.7996/supp-3 Supplemental Information 3 Dynamic of basic morphological parameters per fruit of *S. tonkinensis*.

10.7717/peerj.7996/supp-4 Supplemental Information 4 Dynamics of nutritive and mineral contents based on fresh matter in the pericarp, seed coat, and kernel.

10.7717/peerj.7996/supp-5 Supplemental Information 5 Dynamics of enzyme activities related to oxidation pathway in the pericarp and seed coat.

10.7717/peerj.7996/supp-6 Supplemental Information 6 Raw data containing biological repetition values, means, and standard deviations.

## Additional Information and Declarations

### Data Availability

The following information was supplied regarding data availability: The raw measurements are available in a Supplemental File.
