# Supplementary material for: The nutrient distribution in the continuum of the pericarp, seed coat, and kernel during Styrax tonkinensis fruit development
Source: PeerJ. 2019 Oct 31;7:e7996. doi: 10.7717/peerj.7996 (PMC6825750; doi:10.7717/peerj.7996)
Supplement: Supplemental Information 3 [file peerj-07-7996-s003.docx]

**Table S1:**

**Dynamic of basic morphological parameters per fruit of *S. tonkinensis*.**

|  | **Days after flowering** | | | | | | | | | | | |
| --- | --- | --- | --- | --- | --- | --- | --- | --- | --- | --- | --- | --- |
|  | **30** | **40** | **50** | **60** | **70** | **80** | **90** | **100** | **110** | **120** | **130** | **140** |
| Fruit surface area (mm^2^) | 224.4 ± 44.1 f | 297.4 ± 45.7 e | 334.4 ± 44.3 d | 339.7 ± 70.4 d | 368.0 ± 62.5 c | 374.6 ± 56.4 c | 404.9 ± 73.8 b | 419.4 ± 50.9 ab | 435.4 ± 63.5 a | 439.5 ± 69.2 a | 441.6 ± 44.8 a | 440.9 ± 27.0 a |
| Fruit volume  (mm^3^) | 296.8 ± 91.1 f | 459.1 ± 109.4 e | 550.8 ± 114.4 d | 564.9 ± 185.9 d | 642.9 ± 162.6 c | 661.0 ± 155.2 c | 739.5 ± 224.4 b | 776.8 ± 140.7 ab | 822.8 ± 221.3 a | 834.9 ± 252.3 a | 840.9 ± 130.4 a | 839.0 ± 61.6 a |
| Seed surface area (mm^2^) | 56.2 ± 13.8 f | 87.4 ± 20.4 e | 105.1 ± 19.8 d | 126.6 ± 25.5 c | 143.8 ± 17.8 a | 128.4 ± 17.1 c | 134.7 ± 23.6 bc | 138.5 ± 20.5 ab | 139.1 ± 23.7 ab | 141.7 ± 17.1 ab | 139.5 ± 16.0 ab | 143.2 ± 8.4 a |
| Seed volume  (mm^3^) | 37.4 ± 14.5 f | 73.9 ± 29.0 e | 98.5 ± 27.8 d | 130.9 ± 38.5 c | 159.5 ± 29.9 a | 134.0 ± 26.8 c | 144.0 ± 44.1 bc | 150.0 ± 34.9 ab | 150.5 ± 42.2 ab | 154.9 ± 40.3 ab | 151.1 ± 37.2 ab | 157.3 ± 19.9 a |
