# Supplementary material for: The nutrient distribution in the continuum of the pericarp, seed coat, and kernel during Styrax tonkinensis fruit development
Source: PeerJ. 2019 Oct 31;7:e7996. doi: 10.7717/peerj.7996 (PMC6825750; doi:10.7717/peerj.7996)
Supplement: Supplemental Information 4 [file peerj-07-7996-s004.docx]

**Table S2:**

**Dynamics of nutritive and mineral contents based on fresh matter in the pericarp, seed coat and kernel.**

|  | | **Days after flowering** | | | | | | | | | | | |
| --- | --- | --- | --- | --- | --- | --- | --- | --- | --- | --- | --- | --- | --- |
|  |  | **30** | **40** | **50** | **60** | **70** | **80** | **90** | **100** | **110** | **120** | **130** | **140** |
| Total soluble sugar (mg/g FW) | Pericarp | 45.13 ± 2.04 cde | 44.76 ± 5.23 de | 53.86 ± 2.90 cd | 54.62 ± 2.88 cd | 57.15 ± 1.9 bcd | 37.32 ± 5.55 cde | 52.57 ± 1.91 cd | 48.61 ± 8.18 cde | 58.66 ± 3.92 bc | 69.10 ± 6.94 ab | 76.19 ± 19.67 a | 76.98 ± 3.63 a |
|  | Seed coat | 62.09 ± 1.07 bc | 55.64 ± 3.40 c | 52.22 ± 1.30 c | 58.36 ± 3.96 c | 52.45 ± 3.72 c | 71.20 ± 7.94 a | 33.26 ± 1.75 d | 16.33 ± 0.07 e | 11.08 ± 0.26 e | 11.27 ± 0.75 e | 11.03 ± 0.80 e | 4.34 ± 0.08 f |
|  | Kernel | 121.6 ± 12.45 d | 122.1 ± 8.49 d | 188.8 ± 21.67 b | 204.3 ± 13.34 a | 202.6 ± 13.72 a | 158.4 ± 19.89 c | 124.9 ± 5.92 d | 114.3 ± 0.51 de | 108.1 ± 2.24 e | 112.6 ± 2.81 de | 119.9 ± 2.65 d | 127.8 ± 2.31 cd |
| Total starch (mg/g FW) | Pericarp | 24.88 ± 0.87 cde | 19.91 ± 1.62 def | 20.60 ± 2.20 def | 29.84 ± 3.01 abc | 35.76 ± 6.74 a | 24.47 ± 0.94 cde | 22.42 ± 4.32 def | 17.55 ± 1.91 f | 18.74 ± 2.05 ef | 16.79 ± 4.93 f | 26.24 ± 4.0 bcd | 31.78 ± 5.41 ab |
|  | Seed coat | 8.75 ± 1.00 b | 5.92 ± 0.72 b | 6.88 ± 0.43 b | 11.84 ± 2.11 b | 36.33 ± 7.44 a | 35.89 ± 8.50 a | 23.24 ± 2.75 b | 25.72 ± 3.39 b | 22.80 ± 1.23 b | 25.27 ± 2.56 b | 26.05 ± 3.47 b | 31.61 ± 2.97 b |
|  | Kernel | 121.6 ± 0.41 d | 122.1 ± 0.22 d | 188.8 ± 0.29 b | 204.3 ± 0.30 a | 202.6 ± 0.33 ab | 158.4 ± 0.14 c | 124.9 ± 0.93 a | 114.3 ± 0.57 de | 108.1 ± 0.91 d | 112.6 ± 1.13 d | 119.9 ± 1.50 d | 127.8 ± 1.64 d |
| Total free amino acid (μg/g FW) | Pericarp | 77.23 ± 17.8 cde | 65.42 ± 1.07 de | 76.79 ± 18.0 cde | 151.4 ± 21.0 a | 96.95 ± 2.57 bc | 89.91 ± 12.2 cd | 116.3 ± 21.8 b | 94.59 ± 18.9 bc | 54.27 ± 2.36 ef | 67.57 ± 1.50 de | 57.63 ± 14.92 ef | 35.95 ± 2.72 f |
|  | Seed coat | 54.44 ± 8.62 cde | 61.00 ± 19.8 cd | 58.04 ± 8.37 cde | 89.58 ± 21.4 bc | 110.0 ± 49.03 b | 147.5 ± 21.11 a | 59.72 ± 0.51 cde | 24.58 ± 1.93 e | 57.27 ± 0.90 cde | 38.61 ± 9.01 de | 50.71 ± 13.9 de | 34.36 ± 7.59 de |
|  | Kernel | 98.5 ± 6.44 e | 112.6 ± 21.24 d | 108.1 ± 2.57 | 163.1 ± 18.34 c | 229.1 ± 9.65 ab | 318.5 ± 37.54 a | 161.9 ± 13.08 c | 178.9 ± 26.0 bc | 186.6 ± 21.44 b | 103.9 ± 13.00 e | 110.3 ± 14.54 e | 119.6 ± 24.62 d |
| Total soluble protein (mg/g FW) | Pericarp | 1.25 ± 0.05 bc | 1.22 ± 0.13 bc | 1.21 ± 0.05 bc | 1.27 ± 0.07 bc | 1.35 ± 0.04 ab | 1.38 ± 0.12 ab | 1.15 ± 0.01 c | 1.15 ± 0.07 c | 1.27 ± 0.09 bc | 1.31 ± 0.05 bc | 1.49 ± 0.13 a | 1.29 ± 0.15 bc |
|  | Seed coat | 40.32 ± 1.46 c | 34.91 ± 1.10 d | 38.14 ± 1.44 cd | 51.32 ± 0.47 a | 44.43 ± 3.90 b | 47.17 ± 4.33 b | 18.96 ± 3.22 e | 3.31 ± 0.04 f | 2.70 ± 0.08 f | 2.68 ± 0.10 f | 2.53 ± 0.10 f | 2.80 ± 0.24 f |
|  | Kernel | 53.29 ± 1.49 e | 54.30 ± 4.45 e | 70.49 ± 12.28 d | 78.20 ± 6.83 cd | 92.50 ± 7.90 c | 98.24 ± 1.87 c | 128.5 ± 1.10 b | 129.0 ± 15.03 b | 132.6 ± 15.77 b | 170.1 ± 18.90 a | 179.2 ± 17.37 a | 178.9 ± 18.94 a |
| Potassium (mg/g FW) | Pericarp | 0.98 ± 0.00 e | 1.00 ± 0.06 e | 1.13 ± 0.05 e | 1.32 ± 0.11 de | 2.08 ± 0.06 c | 1.52 ± 0.04 d | 1.66 ± 0.14 d | 2.25 ± 0.21 c | 2.69 ± 0.12 b | 2.96 ± 0.09 ab | 2.72 ± 0.14 b | 3.26 ± 0.31 a |
|  | Seed coat | 0.99 ± 0.02 b | 1.01 ± 0.04 b | 1.26 ± 0.16 a | 1.03 ± 0.03 b | 0.98 ± 0.03 b | 0.86 ± 0.02 c | 1.15 ± 0.11 ab | 1.27 ± 0.03 a | 1.11 ± 0.11 ab | 0.76 ± 0.00 c | 0.47 ± 0.04 d | 0.38 ± 0.13 d |
|  | Kernel | 0.30 ± 0.01 d | 0.31 ± 0.02 d | 0.31 ± 0.18 d | 0.23 ± 0.11 d | 1.69 ± 0.35 b | 1.74 ± 0.03 b | 1.54 ± 0.19 c | 1.56 ± 0.02 c | 1.64 ± 0.12 bc | 1.48 ± 0.05 c | 1.69 ± 0.14 b | 2.64 ± 0.23 a |
| Calcium (mg/g FW) | Pericarp | 8.97 ± 0.25 c | 11.40 ± 3.50 bc | 17.97 ± 5.40 b | 18.22 ± 6.14 b | 23.26 ± 0.24 a | 0.46 ± 0.04 d | 0.48 ± 0.05 d | 13.45 ± 5.07 bc | 18.92 ± 3.88 b | 25.29 ± 0.21 a | 24.41 ± 3.11 a | 18.57 ± 4.62 b |
|  | Seed coat | 4.63 ± 0.39 d | 17.07 ± 0.15 a | 14.21 ± 2.88 b | 16.68 ± 0.99 ab | 14.77 ± 0.36 b | 10.67 ± 1.07 c | 12.87 ± 3.40 bc | 4.08 ± 0.71 d | 1.73 ± 0.03 d | 4.27 ± 0.22 d | 1.63 ± 0.12 d | 2.17 ± 1.05 d |
|  | Kernel | 0.49 ± 0.28 e | 0.21 ± 0.20 e | 9.37 ± 3.42 bc | 10.96 ± 0.23 b | 23.51 ± 3.62 a | 7.51 ± 0.44 d | 7.47 ± 0.94 d | 8.56 ± 0.26 cd | 9.26 ± 1.25 c | 8.75 ± 0.48 cd | 9.66 ± 1.28 bc | 9.57 ± 0.60 bc |
| Magnesium (mg/g FW) | Pericarp | 0.33 ± 0.00 a | 0.36 ± 0.07 a | 0.22 ± 0.06 c | 0.25 ± 0.03 bc | 0.22 ± 0.04 c | 0.09 ± 0.02 d | 0.11 ± 0.01 d | 0.24 ± 0.05 bc | 0.25 ± 0.01 bc | 0.30 ± 0.06 ab | 0.27 ± 0.02 b | 0.28 ± 0.06 b |
|  | Seed coat | 0.22 ± 0.00 d | 0.28 ± 0.01 c | 0.34 ± 0.03 b | 0.39 ± 0.01 a | 0.42 ± 0.01 a | 0.35 ± 0.00 b | 0.42 ± 0.03 a | 0.39 ± 0.01 a | 0.26 ± 0.02 c | 0.22 ± 0.00 d | 0.16 ± 0.02 d | 0.37 ± 0.04 ab |
|  | Kernel | 0.06 ± 0.03 d | 0.08 ± 0.04 d | 0.06 ± 0.04 d | 0.13 ± 0.00 d | 0.57 ± 0.10 c | 0.70 ± 0.04 c | 0.95 ± 0.14 b | 1.03 ± 0.02 a | 1.05 ± 0.10 a | 0.92 ± 0.01 b | 1.01 ± 0.02 a | 1.05 ± 0.16 a |
