# Supplementary material for: The nutrient distribution in the continuum of the pericarp, seed coat, and kernel during Styrax tonkinensis fruit development
Source: PeerJ. 2019 Oct 31;7:e7996. doi: 10.7717/peerj.7996 (PMC6825750; doi:10.7717/peerj.7996)
Supplement: Supplemental Information 5 [file peerj-07-7996-s005.docx]

**Table S3:**

**Dynamics of enzyme activities related to oxidation pathway in the pericarp and seed coat.**

|  | | **Days after flowering** | | | | | | | | | | | |
| --- | --- | --- | --- | --- | --- | --- | --- | --- | --- | --- | --- | --- | --- |
|  |  | **30** | **40** | **50** | **60** | **70** | **80** | **90** | **100** | **110** | **120** | **130** | **140** |
| MDH (U/min/mg protein) | Pericarp | 0.49 ± 0.12 bc | 0.52 ± 0.07 bc | 0.42 ± 0.06 cde | 0.47 ± 0.1 bcd | 0.74 ± 0.17 a | 0.40 ± 0.03 cde | 0.53 ± 0.03 bc | 0.48 ± 0.03 bc | 0.55 ± 0.05 abc | 0.67 ± 0.12 abc | 0.29 ± 0.21 de | 0.23 ± 0.07 e |
|  | Seed coat | 0.10 ± 0.09 b | 0.11 ± 0.04 b | 0.06 ± 0.02 b | 0.06 ± 0.00 b | 0.09 ± 0.05 b | 0.04 ± 0.01 b | 0.43 ± 0.13 a | 0.01 ± 0.00 b | 0.04 ± 0.00 b | 0.01 ± 0.00 b | 0.02 ± 0.02 b | 0.01 ± 0.00 b |
| PGI (U/min/mg protein) | Pericarp | 541 ± 2.7 de | 563 ± 3.8 de | 771 ± 47.3 b | 808 ± 95.4 b | 912 ± 106.3 a | 462 ± 1.6 e | 637 ± 39.7 cd | 696 ± 53.7 cd | 566 ± 13.3 de | 930 ± 74.5 a | 531 ± 34.0 de | 733 ± 30.1 bc |
|  | Seed coat | 34.0 ± 1.8 fg | 63.2 ± 4.4 ef | 99.7 ± 9.3 d | 70.8 ± 13.0 ef | 44.3 ± 29.6 efg | 28.1 ± 10.4 g | 44.3 ± 10.8 efg | 47.9 ± 7.9 efg | 206.8 ± 4.5 a | 236.8 ± 10.4 b | 248.1 ± 39.8 a | 177.2 ± 11.9 c |
| G6PDH (U/min/mg protein) | Pericarp | 6.6 ± 1.11 c | 9.0 ± 0.45 c | 15.0 ± 3.15 b | 20.2 ± 1.63 a | 9.7 ± 3.75 c | 17.8 ± 1.61 ab | 17.7 ± 0.33 ab | 21.6 ± 4.22 a | 20.1 ± 0.60 a | 20.2 ± 0.10 a | 18.6 ± 1.09 ab | 17.4 ± 1.07 ab |
|  | Seed coat | 38.3 ± 0.18 c | 43.1 ± 1.80 ab | 45.6 ± 1.42 a | 39.5 ± 4.53 bc | 36.4 ± 0.30 c | 44.4 ± 2.83 a | 24.4 ± 4.25 d | 28.6 ± 0.61 d | 14.6 ± 1.57 e | 13.2 ± 0.30 e | 6.6 ± 4.19 f | 1.2 ± 0.34 g |
| POD (U/min/g FW) | Pericarp | 1.69 ± 0.19 bc | 1.68 ± 0.13 bc | 1.23 ± 0.02 f | 1.48 ± 0.10 cde | 1.55 ± 0.07 cd | 1.95 ± 0.03 a | 1.35 ± 0.15 def | 1.28 ± 0.03 ef | 1.68 ± 0.02 bc | 1.84 ± 0.07 ab | 1.81 ± 0.23 ab | 1.87 ± 0.12 ab |
|  | Seed coat | 0.10 ± 0.03 d | 0.28 ± 0.12 c | 0.55 ± 0.08 b | 0.71 ± 0.08 a | 0.78 ± 0.05 a | 0.31 ± 0.13 c | 0.06 ± 0.00 d | 0.05 ± 0.02 d | 0.02 ± 0.00 d | 0.01 ± 0.00 d | 0.07 ± 0.01 d | 0.00 ± 0.00 d |
| PPO (U/min/g FW) | Pericarp | 19.4 ± 1.15 ef | 17.5 ± 0.27 f | 14.2 ± 0.11 g | 12.9 ± 0.13 g | 18.9 ± 1.41 ef | 27.3 ± 0.19 c | 17.4 ± 0.45 f | 19.9 ± 1.44 e | 20.6 ± 0.56 e | 32.7 ± 1.79 a | 30.7 ± 2.40 b | 25.1 ± 0.05 d |
|  | Seed coat | 2.83 ± 1.23 fg | 2.17 ± 0.13 g | 6.55 ± 0.18 de | 8.32 ± 0.95 cd | 8.23 ± 1.33 cd | 10.93 ± 1.40 ab | 10.33 ± 1.00 bc | 12.88 ± 2.75 a | 3.65 ± 0.05 fg | 4.27 ± 0.50 fg | 4.62 ± 0.45 ef | 4.52 ± 1.72 ef |
| SOD (U/min/g FW) | Pericarp | 1019 ± 127 d | 1226 ± 152 c | 1372 ± 99 ab | 1259 ± 88 bc | 1310 ± 54 abc | 1355 ± 3 abc | 1354 ± 2 abc | 1353 ± 81 abc | 1348 ± 19 abc | 1407 ± 5 a | 1422 ± 25 a | 1405 ± 20 a |
|  | Seed coat | 20.7 ± 0.99 b | 21.0 ± 3.31 bc | 16.7 ± 0.9 bcd | 10.4 ± 2.51 cd | 14.5 ± 7.3 bcd | 10.2 ± 3.94 d | 69.5 ± 5.99 a | 419.4 ± 14.15 a | 519.7 ± 10.31 a | 539.9 ± 1.92 a | 579.2 ± 8.28 a | 527.0 ± 6.42 a |
| MDA (nmol/g FW) | Pericarp | 6.38 ± 0.12 a | 5.05 ± 0.03 b | 3.03 ± 0.27 d | 2.20 ± 0.07 efg | 3.34 ± 0.25 d | 3.77 ± 0.20 c | 2.03 ± 0.27 fg | 2.56 ± 0.29 e | 2.29 ± 0.06 ef | 1.84 ± 0.17 g | 1.39 ± 0.49 h | 1.79 ± 0.26 gh |
|  | Seed coat | 1.65 ± 0.25 a | 2.01 ± 0.12 a | 0.88 ± 0.1 bcd | 1.18 ± 0.01 bc | 1.33 ± 0.12 b | 1.20 ± 0.3 bc | 0.74 ± 0.3 bcd | 0.55 ± 0.26 bc | 0.60 ± 0.1 cd | 0.81 ± 0.3 bcd | 0.36 ± 0.19 d | 0.82 ± 0.3 bcd |
